# Rectal Transmission of Transmitted/Founder HIV-1 Is Efficiently Prevented by Topical 1% Tenofovir in BLT Humanized Mice

**DOI:** 10.1371/journal.pone.0060024

**Published:** 2013-03-20

**Authors:** Morgan L. Chateau, Paul W. Denton, Michael D. Swanson, Ian McGowan, J. Victor Garcia

**Affiliations:** 1 Division of Infectious Diseases, Department of Internal Medicine, Center for AIDS Research University of North Carolina, Chapel Hill, North Carolina, United States of America; 2 Magee-Womens Research Institute, University of Pittsburgh Medical School, Pittsburgh, Pennsylvania, United States of America; Burnet Institute, Australia

## Abstract

Rectal microbicides are being developed to prevent new HIV infections in both men and women. We focused our *in vivo* preclinical efficacy study on rectally-applied tenofovir. BLT humanized mice (n = 43) were rectally inoculated with either the primary isolate HIV-1_JRCSF_ or the MSM-derived transmitted/founder (T/F) virus HIV-1_THRO_ within 30 minutes following treatment with topical 1% tenofovir or vehicle. Under our experimental conditions, in the absence of drug treatment we observed 50% and 60% rectal transmission by HIV-1_JRCSF_ and HIV-1_THRO_, respectively. Topical tenofovir reduced rectal transmission to 8% (1/12; log rank p = 0.03) for HIV-1_JRCSF_ and 0% (0/6; log rank p = 0.02) for HIV-1_THRO_. This is the first demonstration that any human T/F HIV-1 rectally infects humanized mice and that transmission of the T/F virus can be efficiently blocked by rectally applied 1% tenofovir. These results obtained in BLT mice, along with recent *ex vivo*, Phase 1 trial and non-human primate reports, provide a critically important step forward in the development of tenofovir-based rectal microbicides.

## Introduction

Efficacious biomedical HIV prevention interventions could dramatically reduce the number of new HIV infections globally [1]–[8]. Microbicides (also referred to as topical pre-exposure prophylaxis [topical PrEP]) represent one of several classes (e.g. oral PrEP, treatment-as-prevention) of such interventions currently being developed [9]–[15]. There are multiple reasons why microbicides are attractive as tools for HIV prevention: (i) local administration of an antiretroviral gel at the site of exposure will result in higher drug levels at the intended anatomical location than can be achieved using oral PrEP [16]–[19] while reducing the likelihood of experiencing systemic dosing-associated toxicities [14], [19]; (ii) the reduced toxicity associated with topical microbicides is expected to increase adherence [20]; (iii) microbicides are user controlled [17]; (iv) microbicides are predicted to be cost-effective [21], [22]; (v) topical microbicides can be developed with combinations of viral inhibitors [23]; (vi) an ideal microbicide would be safe and effective in both rectal and vaginal compartments [24]–[26]; and (vii) antiviral microbicides may also protect against viruses other than HIV (e.g. herpes simplex) [27], [28].

All microbicide efficacy clinical trials to date have tested the prevention of vaginal HIV transmission [5], [9], [20], [29]–[36]. However, an important driver of the epidemic in both men and women is HIV transmission resulting from anal intercourse [37]–[44] such that rectal microbicide development is also required [20], [45]–[49]. Proof of concept that administration of an antiretroviral gel rectally can prevent transmission of SIV/SHIV has been demonstrated for tenofovir [50] and MIV-150 [51]. Tenofovir, UC781, and nonoxynol-9 have been tested for safety and acceptability in Phase 1 rectal microbicide clinical trials and, of these three, only tenofovir is being advanced [18]–[20], [52], [53]. Therefore, our *in vivo* preclinical efficacy study in bone marrow-liver-thymus (BLT) humanized mice was designed to determine the efficacy of topical tenofovir for the prevention of rectal HIV-1 transmission.

BLT mice are the experimental platform of choice for this study for several reasons. For example, BLT mice harbor a *de novo* generated human immune system distributed throughout each animal [54]–[76]. In the context of this study, an important characteristic of BLT mice is their susceptibility to rectal HIV-1 transmission [60], [63] due to the presence of human CD4^+^ T cells, macrophages and dendritic cells found throughout BLT mouse intestines, including the rectum [54], [63]. Previously both topical [56] and systemic [59], [60] HIV prevention interventions have been extensively tested in BLT mice for their ability to block vaginal transmission of HIV-1. The results obtained from these studies were highly predictive of the clinical trial outcomes [9], [13], [56], [59], [60], [77].

An important and novel aspect of this study is the use of a MSM-derived transmitted/founder (T/F) virus [78]. Typically only one or a few virions (defined as the T/F viruses) are responsible for a mucosal transmission event in humans making T/F viruses physiological relevant for *in vivo* efficacy studies of HIV prevention interventions [79], [80]. BLT mice were treated rectally with topical 1% tenofovir and then rectally inoculated with HIV-1_JRCSF_, a well characterized low passage primary isolate, or the T/F virus HIV-1_THRO_. We found that rectal transmission of both viruses was efficiently prevented by topical tenofovir.

## Materials and Methods

### Preparation of BLT Mice and Characterization of Human Reconstitution

BLT mice were prepared essentially as previously described [54]–[61], [63], [76]. Briefly, thy/liv implanted [81] and preconditioned NOD/SCID-gamma chain null (NSG) mice (Jackson Laboratories, Bar Harbor, ME) were transplanted with autologous human fetal liver CD34^+^ cells (Advanced Bioscience Resources, Alameda, CA) and monitored for human reconstitution in peripheral blood by flow cytometry [59], [61], [63]. Mice were maintained at the University of North Carolina at Chapel Hill Division of Laboratory Animal Medicine in accordance with protocols approved by the Institutional Animal Care and Use Committee.

### Topical Application of Tenofovir and Rectal Exposure of BLT Mice to HIV-1

Stocks of HIV-1_JRCSF_ [82] and HIV-1_THRO_ [78] were prepared and titered as we have previously described [57], [83]. Mice were exposed rectally using 0.6 µg p24 of HIV-1_JRCSF_ (4×10^6^ TCIU, tissue culture infectious units) and 0.7 µg p24 of HIV-1_THRO_ (5×10^6^ TCIU). Topical tenofovir consisted of 1% tenofovir (PMPA; 9-(2-phosphonyl-methoxypropyly)-adenine) in PBS. The vehicle (placebo) control was PBS.

**Figure 1:**
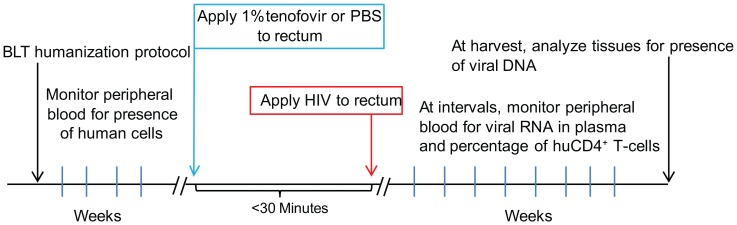
Experimental design and timeline. BLT mice were utilized to determine the efficacy of topically applied tenofovir to prevent rectal HIV-1 transmission. Rectal HIV-1 exposures were performed within 30 minutes following rectal application of 1% tenofovir. Plasma viral load and real time PCR amplification of tissue associated viral DNA were used as HIV-1 detection strategies to determine whether peripheral blood samples collected at the indicated times and tissues collected at harvest contained HIV-1.

The exposure timeline (Figure 1) consisted of rectal application of vehicle or of 1% tenofovir less than 30 minutes prior to rectal application of virus. Rectal exposures with HIV-1_JRCSF_ and HIV-1_THRO_ were performed essentially as previously described [60], [63] except that all the mucosal exposures were carried out atraumatically and without simulated rectal intercourse [84]. All rectal applications of vehicle or inhibitor as well as virus were performed while mice were anesthetized [60], [63]. After viral exposure, mice were returned to their housing to recover and were then monitored longitudinally for evidence of HIV-1 infection as indicated below.

### Analysis of HIV-1 Infection of BLT Mice

Infection of BLT mice with HIV-1 was monitored at the indicated time intervals in peripheral blood by determining plasma levels of viral RNA using real time PCR (limit of detection 750 copies/ml) [55], [56] and by monitoring CD4^+^ T cell percentages by flow cytometry [59], [60]. At necropsy, tissues were harvested and mononuclear cells isolated as previously described [54], [56], [59], [61], [63]. Mononuclear cells were washed, enumerated and tested using real time PCR for the presence of HIV-1 DNA (limit of detection 10 copies) [56], [57], [59], [60].

Sequence analysis was performed on plasma RNA samples in the sole case of breakthrough infection of a tenofovir-treated, HIV-1_JRCSF_-exposed BLT mouse. The entire reverse transcriptase gene from plasma HIV-1 RNA amplification products was sequenced. No resistance mutations in reverse transcriptase were present [85]–[88].

### Statistics

All statistical analyses (alpha level: 0.05) were performed using Prism v. 5 (Graph Pad Software). Kaplan-Meier plots indicate the percentage of animals that are HIV-1 positive in the peripheral blood at each time point analyzed. Power analysis calculation for experimental group sample sizes were determined as previously described [89], [90]. Briefly, we assumed 50 and 65% variance in transmission between our experimental groups for HIV-1_JRCSF_ and HIV-1_THRO_, respectively. In the case of each viral isolate, the chosen sample sizes were determined to have 90% power to detect statistically significant differences via log rank test analysis in the treatment arm versus the vehicle arm.

## Results

### Baseline Characterization of BLT Mouse Human PBMC Reconstitution

This study was designed to determine the *in vivo* efficacy of topical tenofovir for the prevention of rectal HIV-1 transmission. Prior to HIV-1 exposure of the BLT mice, their peripheral blood was characterized by flow cytometry to confirm reconstitution with human cells. All BLT mice used herein (n = 43) had high peripheral blood reconstitution levels of human lymphoid (CD45^+^) cells (67% mean ±17 SD) and human CD4^+^ T cells (80% mean ±6 SD) (Summarized in Tables 1 and 2).

**Table 1:** BLT mice used to test the efficacy of topical tenofovir to prevent rectal **HIV-1_JRCSF_** transmission.*

	Mouse	% human CD45^+^ in PBat exposure	% hCD45^+^ hCD3^+^ hCD4^+^in PB at exposure	Tissue Cell associatedviral DNA^∧^	HIV Status
**Topical Tenofovir**	J01	78	87	B, S, O, LN	Neg
	J02	69	86	B, S, O, LN,	Neg
	J03	67	79	B, S, O, LN	Neg
	J04	39	83	B, S, O, LN	Neg
	J05	78	67	**B,** S, **O, LN**	**Pos**
	J06	54	84	B, O, LN	Neg
	J07	69	71	B, S, LN	Neg
	J08	73	68	BM, S, O, LN	Neg
	J09	86	69	ND	Neg
	J10	65	88	BM, S, O, LN	Neg
	J11	79	86	B, S, O	Neg
	J12	80	86	BM, S, O, LN	Neg
Mean (+/− SD)		70% (+/−13)	80% (+/−8)		
**Vehicle**	J13	73	88	**B, S, O, LN**	**Pos**
	J14	52	79	ND	Neg
	J15	32	85	ND	Neg
	J16	84	73	**B,** S, **O,** LN,	**Pos**
	J17	68	86	ND	**Pos**
	J18	56	85	ND	Neg
	J19	31	79	B, S	**Pos**
	J20	83	72	B, O, S,	Neg
	J21	61	71	**S, O, LN,**	**Pos**
	J22	71	83	ND	Neg
	J23	79	88	B, S, O, LN	Neg
	J24	62	84	B, S, O, LN	Neg
	J25	72	84	ND	Neg
	J26	60	80	**S, O, LN**	**Pos**
	J27	75	82	B, S, O, LN	Neg
	J28	76	88	**B, S, O, LN**	**Pos**
	J29	77	87	ND	**Pos**
Mean (+/− SD)		65% (+/−16)	82% (+/−6)		

* The data shown in the table includes analyses performed on both infected and uninfected mice with the text in bold used to highlight that HIV-1 was found in the indicated tissues.

ˆAbbreviations: B – bone marrow; LN –lymph nodes; ND - not done; Neg – negative; O – thymic organoid; PB – peripheral blood; Pos – positive; and S – spleen.

**Table 2:** BLT mice used to test the efficacy of topical tenofovir to prevent rectal **HIV-1_THRO_** transmission.*.

	Mouse	% human CD45+in PB at exposure	% hCD45+ hCD3+ hCD4+in PB at exposure	Tissue Cell associatedviral DNA^∧^		HIV Status
**Topical Tenofovir**	T01	56	83	B, S, O, LN		Neg
	T02	81	81	B, S, O, LN		Neg
	T03	82	77	B, S, O, LN		Neg
	T04	87	82	B, S, O, LN		Neg
	T05	24	80	B, S, O, LN		Neg
	T06	29	80	B, S, O, LN		Neg
Mean (+/− SD)		60% (+/−28)	81% (+/−2)			
**Vehicle**	T07	61	82	**B, S, O,**		**Pos**
	T08	85	81	B, S, O, LN		Neg
	T09	70	76	**B, S, O**		**Pos**
	T10	86	78	**B, S, O**		**Pos**
	T11	42	83	**B, S, O**		**Pos**
	T12	56	77	ND		Neg
	T13	83	75	B, S, O,LN		Neg
	T14	73	78	ND		**Pos**
Mean (+/− SD)		70% (+/−16)	79% (+/−3)			

* The data shown in the table includes analyses performed on both infected and uninfected mice with the text in bold used to highlight that HIV-1 was found in the indicated tissues.

ˆAbbreviations: B – bone marrow; LN –lymph nodes; ND - not done; Neg – negative; O – thymic organoid; PB – peripheral blood; Pos – positive; and S – spleen.

### Topical Tenofovir Prevents Rectal HIV-1_JRCSF_ Transmission

**Figure 2:**
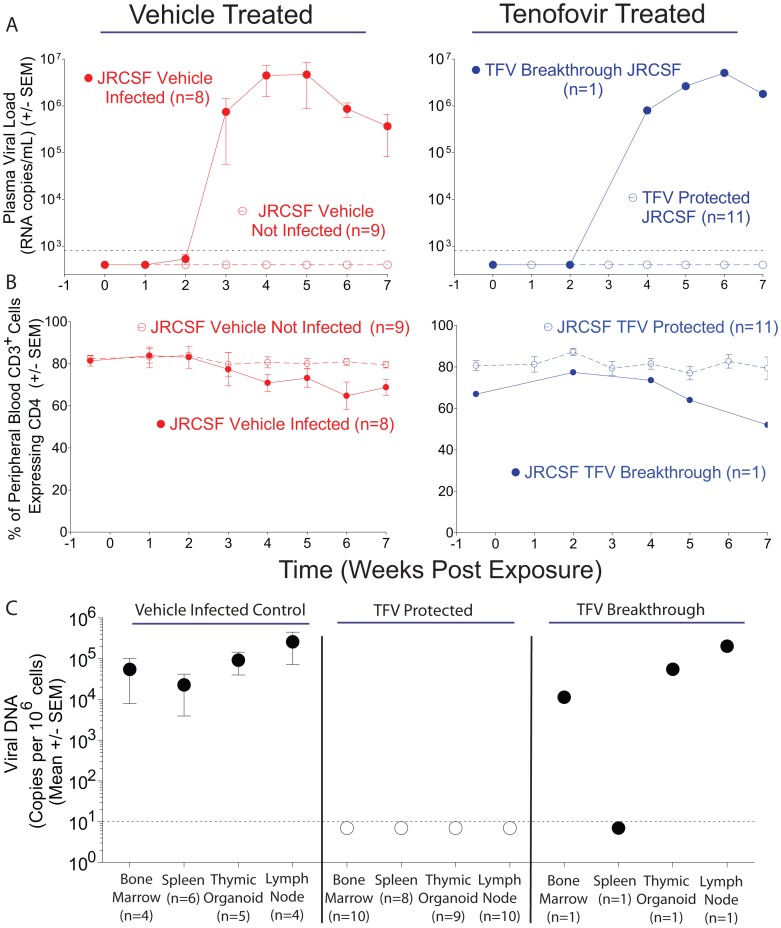
Analysis of peripheral blood and tissues for the presence of HIV-1_JRCSF_ after rectal exposure in the presence or absence of topical tenofovir. (A–B) Longitudinal analyses of peripheral blood plasma viral RNA (A) and the percentage of peripheral blood CD3^+^ T cells also expressing CD4 (B) are presented for vehicle (left) and topical tenofovir (right) -treated BLT mice exposed rectally to HIV-1_JRCSF_. (C) Real-time PCR analysis of tissues for presence or absence of HIV-1 DNA. Thin dashed lines represent the limit of detection for the respective assays. Error bars indicate standard error of the mean. Open symbols are used to depict data from HIV negative mice and closed symbols are used to depict data from HIV positive mice.

A total of 29 mice were exposed to HIV-1_JRCSF_, a CCR5-tropic virus that has been well characterized for its mucosal infection of BLT mice [56], [57], [59], [60], [63], [75], [76]. Seventeen mice received vehicle and 12 mice received topical tenofovir (Figure 2; Table 1). Following viral exposure, peripheral blood from the BLT mice was sampled weekly for the presence of HIV-1 RNA (Figure 1). Eight of the 17 mice in the control arm of the experiment were infected as determined by the presence of viral RNA in plasma (Figure 2A). In contrast, 11 of 12 topical tenofovir treated mice were consistently negative for the presence of plasma viral RNA (Figure 2A). One tenofovir treated mouse was found to have a ‘breakthrough’ infection with readily detectable plasma viral RNA (Figure 2A). No tenofovir resistant mutations from this breakthrough virus were identified when the entire reverse transcriptase gene was sequenced. Over the course of this experiment, we also monitored the levels of CD4^+^ T cells in peripheral blood. The breakthrough infection mouse and the infected vehicle control mice maintained similar peripheral blood CD4^+^ T cell levels to the HIV-1 negative mice (Figure 2B), as we have previously observed with this CCR5-tropic HIV-1 isolate in BLT mice [59], [60].

**Figure 3:**
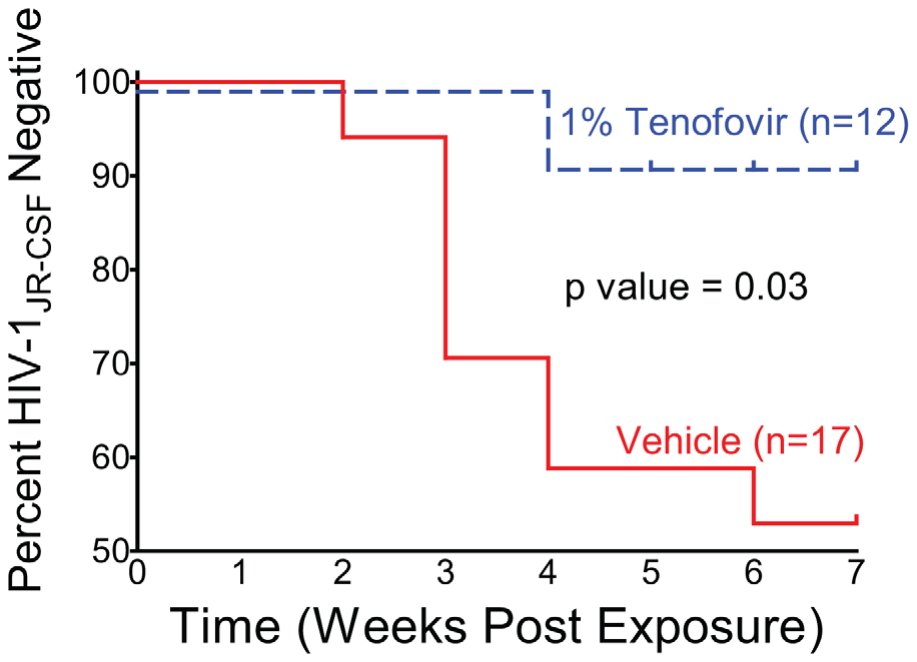
Topical tenofovir prevents rectal HIV-1_JRCSF_ transmission in BLT mice. Kaplan-Meier plot indicates the time to peripheral blood conversion following rectal HIV-1_JRCSF_ exposure in BLT mice pretreated with either vehicle or topical tenofovir. Log-rank (Mantel Cox) analysis reveals a statistically significant difference in rectal HIV-1_JRCSF_ transmission between the vehicle and topical tenofovir arms.

Prior to defining topical tenofovir treated BLT mice as protected from rectal HIV-1 transmission, we tested tissues harvested from these mice for the presence of cell-associated HIV-1 DNA. All mice without plasma viral RNA were also found to be negative for viral DNA in all tissues evaluated (e.g. bone marrow, spleen, human thymic organoid and lymph nodes) confirming the lack of HIV-1 transmission in these animals (Figure 2C; Table 1). The HIV status and time to plasma viremia were then combined to generate a Kaplan-Meier plot of the protection from rectal HIV transmission provided by either the vehicle or topical tenofovir (Figure 3). Log rank analysis (p = 0.03) confirmed that topical tenofovir prevents rectal HIV-1_JRCSF_ transmission in BLT mice.

### Rectal Transmission of Transmitted/Founder HIV-1_THRO_ is Prevented by Topical Tenofovir

**Figure 4:**
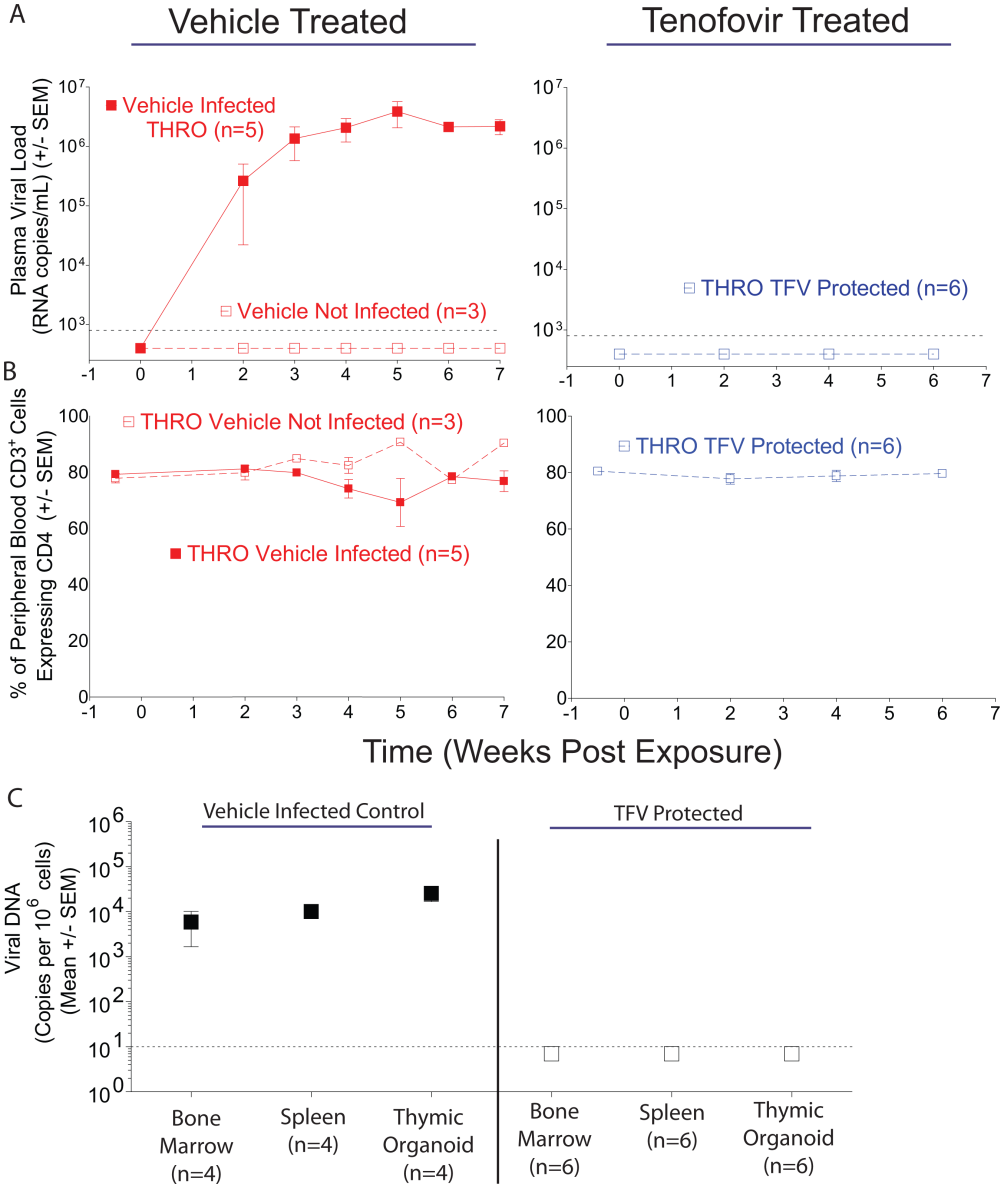
Analysis of peripheral blood and tissues for the presence of HIV-1_THRO_ after rectal exposure in the presence or absence of topical tenofovir. (A–B) Longitudinal analyses of peripheral blood plasma viral RNA (A) and the percentage of peripheral blood CD3^+^ T cells also expressing CD4 (B) are presented for vehicle (left) and topical tenofovir (right) -treated BLT mice exposed rectally to HIV-1_THRO_. (C) Real-time PCR analysis of tissues for presence or absence of HIV-1 DNA. Thin dashed lines represent the limit of detection for the respective assays. Error bars indicate standard error of the mean. Open symbols are used to depict data from HIV negative mice and closed symbols are used to depict data from HIV positive mice.

HIV-1_THRO_ is a CCR5-topic, MSM-derived T/F virus [78]. A total of 14 BLT mice were exposed rectally to HIV-1_THRO_ (Figure 4). Eight mice received vehicle and six mice received tenofovir. Five of the mice receiving vehicle were infected as determined by the presence of plasma virus RNA (Figure 4A). In contrast, none of the tenofovir treated BLT mice (0/6) exposed rectally to HIV-1_THRO_ exhibited plasma viremia (Figure 4A). In addition to plasma viremia, we also monitored the levels of human CD4^+^ T cells in the peripheral blood of all the HIV-1_THRO_ exposed mice. The levels of human CD4^+^ T cells in the infected mice did not change throughout the course of infection (Figure 4B).

**Figure 5:**
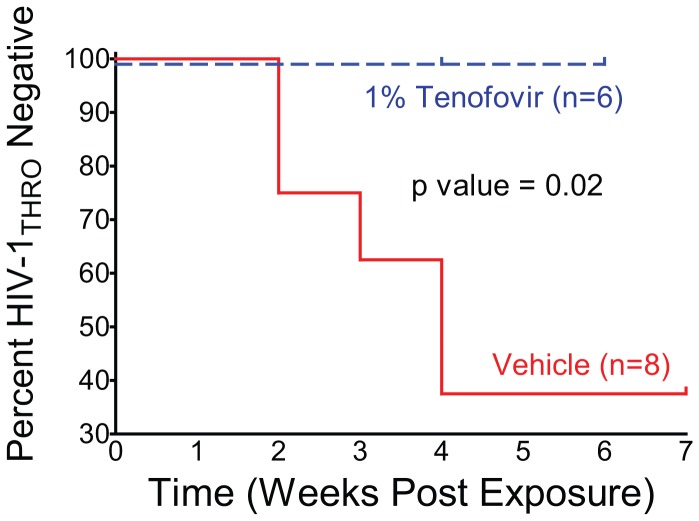
Topical tenofovir prevents rectal transmission of HIV-1_THRO,_ a T/F virus_,_ in BLT mice. Kaplan-Meier plot indicates the time to peripheral blood conversion following rectal HIV-1_THRO_ exposure in BLT mice pretreated with either vehicle or topical tenofovir. Log-rank (Mantel Cox) analysis reveals a statistically significant difference in rectal HIV-1_THRO_ transmission between the vehicle and topical tenofovir arms.

To confirm the lack of HIV-1 infection of the tenofovir treated mice we used real time PCR to determine the presence of cell-associated HIV-1 DNA in tissues obtained from these mice. None of the mice treated with tenofovir had detectable levels of viral DNA in any of the tissues examined (Figure 4C; Table 2). In contrast, the presence of viral DNA in tissues from infected animals was readily confirmed (Figure 4C; Table 2). Log rank analysis of these results presented in a Kaplan-Meier plot (Figure 5) revealed that topical tenofovir administered prior to exposure to BLT mice prevents rectal transmission of the physiologically relevant T/F virus, HIV-1_THRO_ (p = 0.02).

## Discussion

Mucosal infection after sexual intercourse is the most common route of HIV-1 transmission worldwide which makes the cervicovaginal and rectal mucosa the two most important anatomical sites for viral exposure [91]. Receptive anal intercourse has the highest risk of HIV-1 infection and accounts for most new infections in the US [92], [93]. Nevertheless, the vast majority of past and ongoing clinical trials for HIV prevention using topical microbicides have focused on preventing vaginal HIV-1 acquisition [5], [9], [20], [29]–[36]. The formulation of tenofovir 1% gel used in the RMP-02/MTN-006 Phase 1 rectal safety study was the same formulation used vaginally in the CAPRISA 004 trial [9], [19]. Unfortunately, there was a significant increase in gastrointestinal adverse events seen in the RMP-02/MTN-006 study, possibly due to the hyperosmolar nature of the gel [19], [20]. We therefore elected to evaluate the efficacy of tenofovir directly, in the absence of any type of gel, to make a clear determination of the potential efficacy of tenofovir for the prevention of rectal HIV transmission. Our study supports the choice of tenofovir as an appropriate active pharmaceutical ingredient around which a specifically engineered microbicide can be designed for rectal [18]–[20] or dual compartment use [25], [26].

Our goal was to evaluate the *in vivo* efficacy of a rectal microbicide candidate for inclusion into a rectal microbicide to prevent HIV-1 acquisition. We focused on rectal HIV transmission because this route of virus spread continues to be a major contributor to the number of men and women becoming infected with HIV [37]–[43]. We chose a topical intervention because of the many potential benefits associated with this drug delivery route [14], [17], [19]–[28]. BLT mice were chosen as the experimental platform for this evaluation because previous studies have shown that FDA approved drugs prevent mucosal HIV transmission of the human primary virus isolate HIV-1_JRCSF_ in this model [56], [59], [60]. Here when BLT mice were pretreated with topical tenofovir (or vehicle) and then rectally exposed to HIV-1_JRCSF_, we found that topical tenofovir efficiently prevents rectal transmission of HIV-1_JRCSF_ (Figures 2 and 3; Table 1).

To extend and expand on this observation we also evaluated the protective effect of tenofovir using a second virus, HIV-1_THRO_. HIV-1_THRO_ is a MSM-derived T/F virus and therefore its evaluation in the context of rectal transmission is of significant relevance [78]. T/F viruses represent the one or few founder viruses that undergo amplification in local T cells and subsequent systemic dissemination after mucosal exposure [78]–[80], [94]. These T/F viruses use CCR5 as a coreceptor for entry and replicate poorly in monocyte/macrophages relative to T cells [78]. Despite their intrinsic relevance, T/F viruses have not been previously used for *in vivo* transmission studies in animal models. We found that HIV-1_THRO_ transmits rectally in BLT mice and that its transmission can be efficiently prevented by pretreatment with rectally applied tenofovir (Figures 4 and 5; Table 2).

Analysis of the data from two HIV-1 isolates indicates that 1 of 18 BLT mice became infected despite treatment with topical 1% tenofovir prior to rectal HIV-1 exposure, while 13 of 25 vehicle treated BLT mice became infected (p = 0.002 Fisher’s exact test) (Tables 1 and 2). In an *in vivo* study using non-human primates (NHP), 2 of 6 macaques became infected despite treatment with topical 1% tenofovir 15 minutes prior to rectal SIV exposure, while 3 of 4 vehicle treated macaques became infected [50]. The conclusion reached by the authors of the macaque study and our conclusion of the study presented here are the same – topical tenofovir can inhibit rectal transmission of SIV [50], primary HIV-1 (Figure 3) and T/F HIV-1 (Figure 5).

Topical microbicides are of significant interest in HIV prevention because they achieve high local drug concentrations capable of preventing HIV transmission with reduced risk for toxicity [14], [17], [19]. The *in vivo* preclinical efficacy data presented here together with previous data from NHP [50] show that topical tenofovir can efficiently block rectal transmission. The incorporation of a physiologically relevant T/F HIV-1 into this study of rectal HIV prevention increases its translational value. The results presented here show the importance of animal models for the evaluation of HIV-1 prevention strategies and demonstrate the potential for efficacy of tenofovir-based rectal microbicides in humans. Future studies will leverage the results from this work and the BLT model to perform dose-ranging tenofovir studies, evaluate rectal-specific gel formulations containing tenofovir and evaluate other topical rectal microbicide agents for efficacy.
